# Niemann-pick disease with visceral and pulmonary involvement in a resource limited setting: A rare case report

**DOI:** 10.1016/j.radcr.2024.11.021

**Published:** 2024-12-21

**Authors:** Eyasu Wakjira Korsa, Samuel Sisay Hailu, Hewan Asfaw, Hanna Damtew, Daniel Zewdneh Solomon

**Affiliations:** a Department of Radiology, Wudassie Diagnositic Center, Addis Ababa, Ethiopia; b Department of Radiology, Addis Ababa University, College of Health Sciences, Addis Ababa, Ethiopia; c Department of Pediatrics and Child Health, Addis Ababa University, College of Health Sciences, Addis Ababa, Ethiopia; d Children's Hospital of Philadelphia, Philadelphia, Pennsylvania, United States

**Keywords:** Niemann-Pick disease, Lysosomal storage disease, Foam cells, Acid sphingomyelinase deficiency

## Abstract

Niemann-Pick disease (NPD) is an autosomal recessive disease caused by deficient lysosomal enzyme or faulty cholesterol transport. A 9-year-old male patient presented with 6 years of abdominal swelling, previously treated as tuberculosis. He exhibited hepatosplenomegaly, delayed growth, and pancytopenia. Imaging revealed hepatosplenomegaly, a focal splenic infarct, diffuse interstitial septal thickening, and ground glass opacities in the lungs, raising suspicion of a storage disease. Further biopsies, of the bone marrow and liver, revealed the presence of foam cells with abundant multivacuolated cytoplasm and central round nuclei, suggesting NPD. Genetic testing and specific enzyme activity tests are unavailable in our setting. This case highlights the diagnostic challenges of rare disease in resource-limited settings, often mistaken for more common conditions like tuberculosis and lymphoma.

## Introduction

Niemann-Pick disease is a historic eponym corresponding to 2 distinct and rare inherited autosomal recessive diseases that belong to a group of lysosomal storage diseases. Lysosomal storage diseases develop whenever lysosomes fail to break down complex molecules or fail to release their degradation products [1].

The incidence of NPD is variable depending on the subtypes. Subtypes A and B combined have been estimated to be 1 in 250,000 live births, NPD subtype type C is 1 in 150,000 live births [1].

The underlying etiology of NPD can be divided into 2 distinct entities. The first one is deficiency acid sphingomyelinase enzyme resulting from mutations in the sphingomyelin phosphodiesterase 1 [SMPD1] gene encompassing Type A and B. The second one is a defect in cellular cholesterol trafficking resulting from a mutation in either NPC1 or NPC1 genes, responsible for Type C [[2], [3], [4], [5]]

The deficiency of acid sphingomyelinase enzymes is the cause of Type A and B NPD. This enzyme is needed to break down sphingomyelin (SM) into ceramide and phosphocholine. As a result, SM accumulates in the lysosomes of macrophages of the tissues of the reticuloendothelial system, the lungs, and the brain resulting in hepatosplenomegaly, cytopenia, lung disease, and neurologic symptoms. These lipid-laden macrophages have a large size, foamy and vacuolated appearance due to the deposition of SM. These cells are also called Niemann-Pick cells or foam cells [2,6].

NPD subtype A is seen in infants and affects the central nervous system and visceral organs hence also called infantile neurovisceral form. Most affected children die before the age of three. NPD type B has a less severe disease progression. Unlike Type A, visceral symptoms are seen to a variable degree with minimal neurologic disease. Hepatosplenomegaly, thrombocytopenia, and interstitial lung disease are the most common findings associated with Type B NPD. NPD type C shows a wide phenotypic variability which is witnessed even among familial NPD type C cases suggesting multiple contributing factors. In NPD type C, the manifestation in the perinatal period and infancy are predominantly visceral (including liver and lung disease), while in childhood and adulthood, the presentation is dominated by neurologic manifestations, such as ataxia, dystonia, supranuclear gaze palsy, and psychiatric involvement(mainly in adults). The classic presentation is in mid-childhood and adolescence [[3], [4], [5], [6], [7]].

The presence of Niemann-Pick cells in the tissues such as the reticuloendothelial system or lungs, although not confirmatory, are indicative of the acid sphingomyelinase deficiency, [ASMD]. Reduced activity ASM in the circulating leukocytes or cultured skin fibroblast is confirmatory of types A and B ASMD. Genetic sequencing for the specific mutations is also confirmatory [1,2].

Authors present a case of a child with hepatosplenomegaly and pulmonary abnormalities. Due to the rarity of the disease, limited experience of the practicing physicians, and limited diagnostic tools, most patients in resource-limited settings such as ours are usually misdiagnosed and subjected to unnecessary treatment. Our patient was previously misdiagnosed and treated as a case of pulmonary tuberculosis before presenting to our hospital. We believe that was because of similarities in presentation and the high burden of tuberculosis in our setting. This report highlight importance of maintaining a high index of suspicion for such rare diseases to avoid misdiagnosing them as more common disease encountered in daily clinical practice. Furthermore, it underscores how lack of diagnostic tools and treatment for rare diseases affect patient care in countries with low and middle-income countries.

## Case presentation

A 9-year-old male patient presented to our hospital with abdominal swelling of 6 years. The swelling was getting worse in the last 1 year and 2 months before the current presentation. He had a cough, night sweats, easy fatigability, and a significant but unquantified amount of weight loss. He was treated at an outside hospital for tuberculosis with no significant improvement. There is no family history of a similar illness.

Physical examination showed normal vital signs and a grossly distended abdomen with palpable liver measuring 10 cm below the right costal margin and a total liver span of 15cm. The spleen was also significantly enlarged measuring 18cm along the line of growth. Neurologic examination was normal. Anthropometric findings were weight 24kg, Height 109cm, BMI- 20.2kg/m2, and length for age is less than -3 z score.

Complete blood count was suggestive of pancytopenia with white blood cell count of 3360/µL, hemoglobin of 9gm/dL and platelet count of 92000/µL. He had elevated liver transaminases AST = 200 units/L (reference range = 15–40 units/L), ALT = 116 units/L (reference range = 4–36 units/L), and ALP=124 units/L (reference range = 15–40 units/L). Genexpert was negative twice. Viral markers and provider-initiated counseling and testing (PICT) for HIV are nonreactive.

**Fig. 1:**
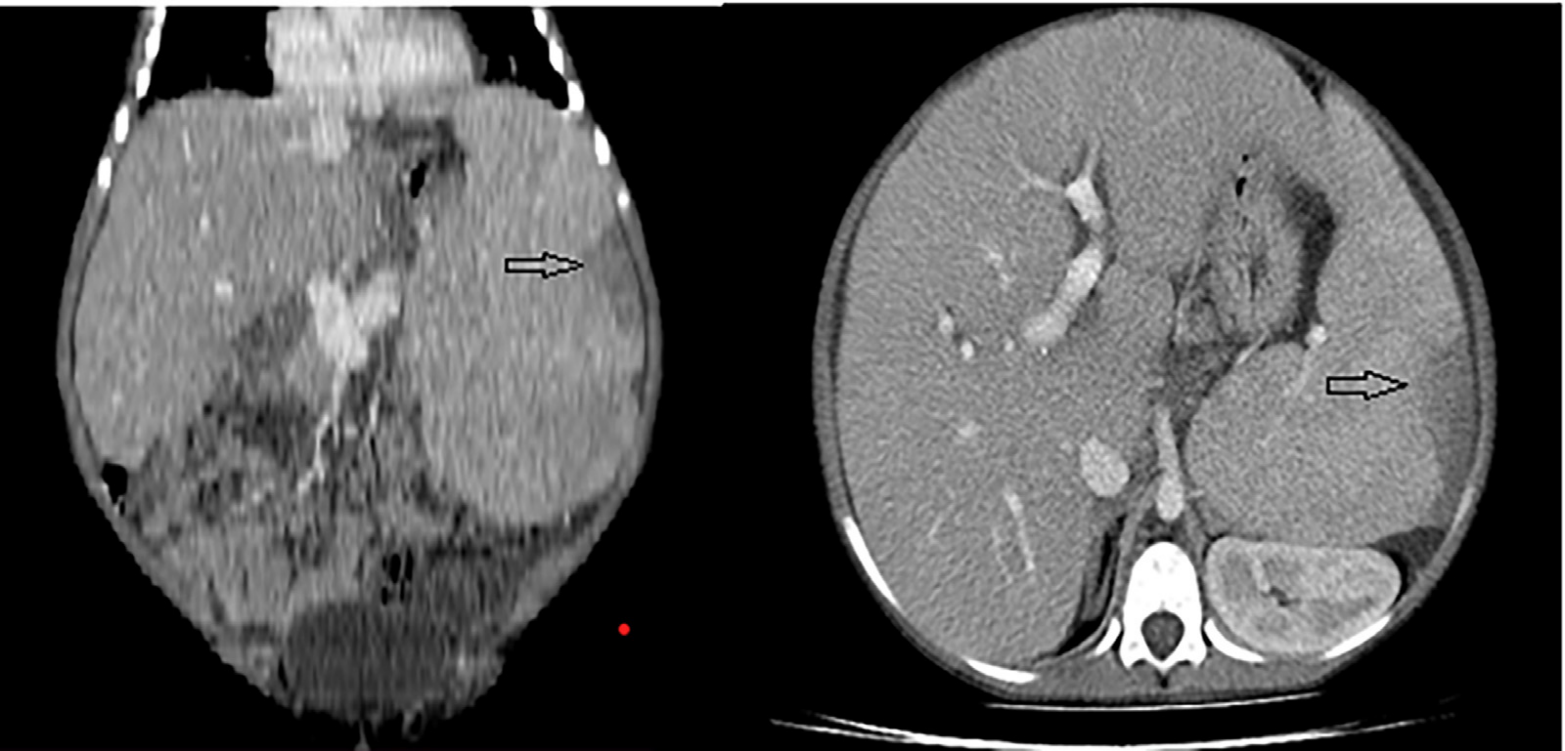
Hepatosplenomegaly and splenic infarct. Coronal and axial postcontrast CT scan of the abdomen showing hepatosplenomegaly and wedge-shaped splenic infarct (Arrow).

Abbreviations: NPD, Niemann–Pick Disease; ASM, acid sphingomyelinase; ILD, interstitial lung disease; SMPD1, sphingomyelin phosphodiesterase 1.

Abdominal ultrasound and pre and postcontrast abdominal CT showed huge hepatosplenomegaly with focal wedge-shaped splenic infarction (Fig. 1).

**Fig. 2:**
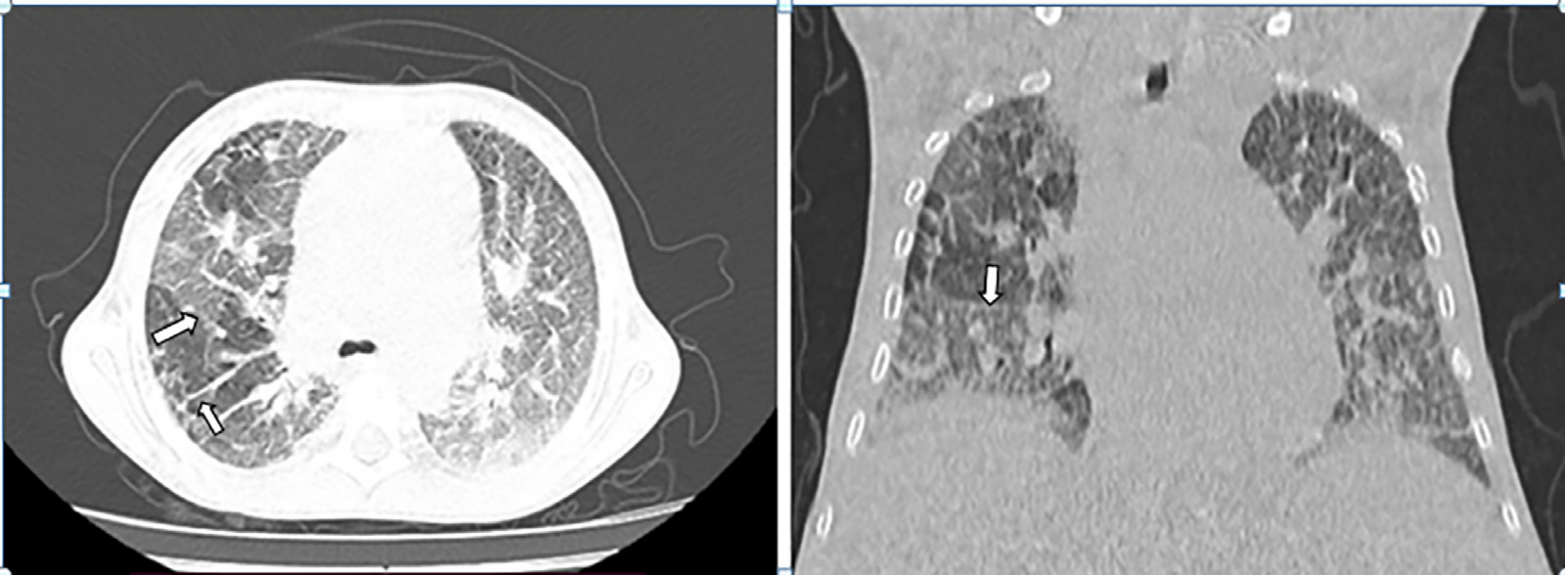
Interstitial lung disease. Axial and coronal chest CT scan showing diffuse septal thickening and ground glass opacities (Arrows).

A high-resolution chest CT showed diffuse interstitial septal thickening and ground glass opacities with no lymphadenopathies suggesting interstitial lung disease (ILD) (Fig. 2).

Based on the imaging findings, a radiologic diagnosis of Gaucher's disease was suggested.

**Fig. 3:**
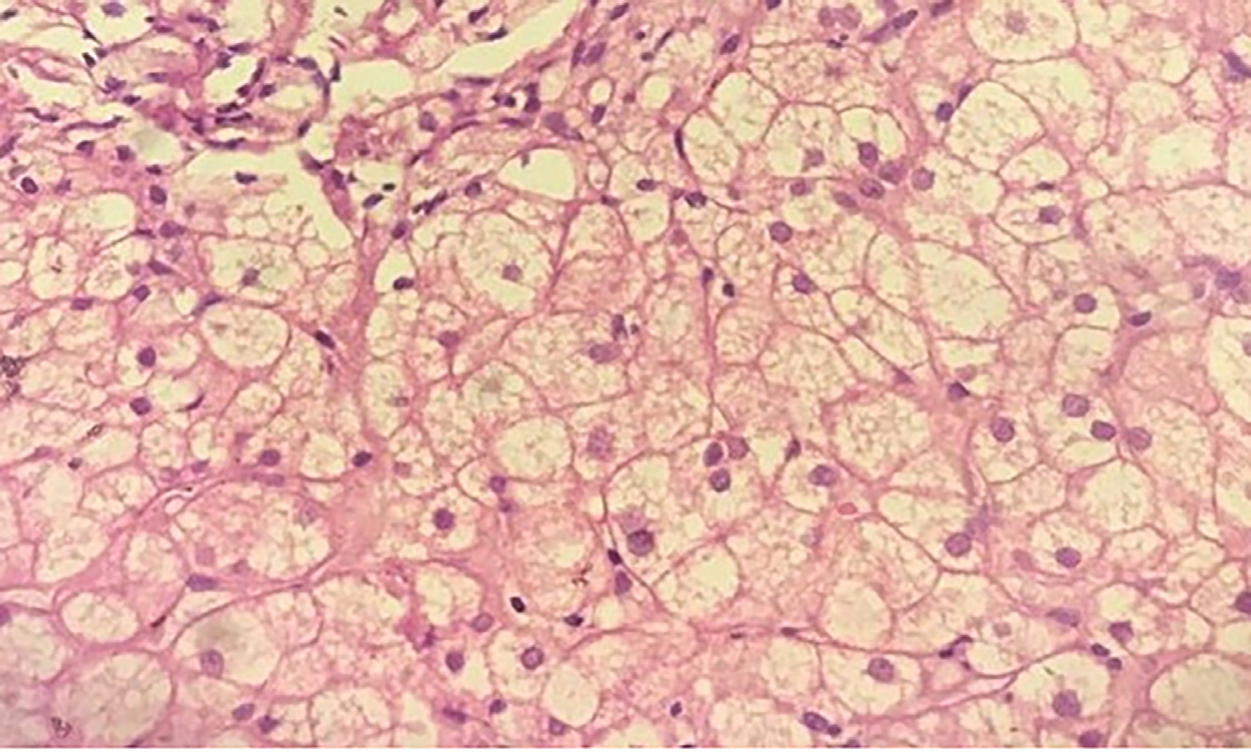
Hepatic foam cells. The histologic section from the liver shows mainly sheets of round to polygonal multivacuolated foam cells having abundant multivacuolated cytoplasm and central round nuclei, with no normal liver parenchyma (Hematoxylin and Eosin stain; 400× magnification).

**Fig. 4:**
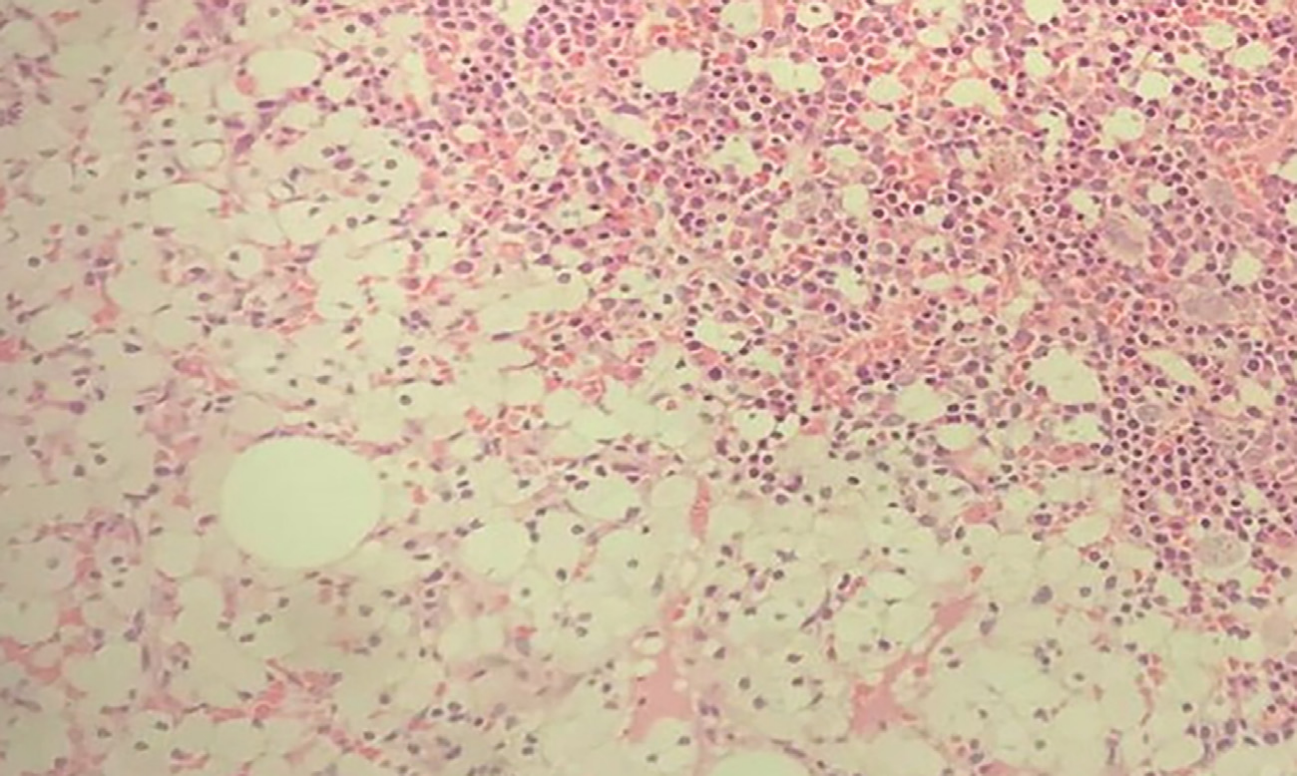
Polygonal marrow macrophages with vacuolated cytoplasm. Histologic section from bone marrow shows hematopoietic marrow elements and areas of sheets of round to polygonal macrophages having vacuolated cytoplasm and central round nuclei. (Hematoxylin and Eosin stain; 100× magnification).

Subsequently, liver and bone marrow biopsies were performed. Histopathology of the liver sample showed sheets of round to polygonal multivacuolated foam cells having abundant multivacuolated cytoplasm and central round nuclei, with no normal liver parenchyma identified (Fig. 3). Histologic sections of the bone marrow aspirate showed hematopoietic marrow elements and areas of sheets of round to polygonal macrophages having vacuolated cytoplasm and central round nuclei (Fig. 4). Based on these histologic findings, a diagnosis of Niemann-Pick disease was made.

## Case discussion

Our case is a 9-year-old male child who came to our radiology department for nonspecific hepatosplenomegaly. Imaging findings on abdominal ultrasound and CT were hepatosplenomegaly and wedge-shaped splenic infarction. In addition to that high resolution chest CT showed interstitial lung disease (ILD). Based on these imaging findings, our initial radiologic diagnosis was Gaucher's Disease.

Having identified hepatomegaly and huge splenomegaly, associated with cytopenia on complete blood count, it was decided to perform a bone marrow and liver biopsy. Histopathologic findings of multivacuolated foam cells with abundant cytoplasm and central round were the suggestive clues for lysosomal storage disease making infectious, hematologic, and tumor conditions less likely. Gaucher's disease can present with hepatosplenomegaly, cytopenia, and ILDs. But in Gaucher's disease, bone pain and bone lesions are prominent [1]. Also hallmark of Gaucher's disease on histopathology are macrophages that have a characteristic wrinkled-paper appearance, resulting from intracytoplasmic substrate deposition [[7], [8]]. Even if our patient had hepatosplenomegaly and evidence of ILD, the absence of bone involvement and the presence of multivacuolated foam cells were against Gaucher's disease.

ASMD is characterized by its variable clinical presentation. ASMD presents in childhood with hepatosplenomegaly, cytopenia, and slowed bone growth [4]. Neurologic symptoms are seen less frequently in Type B [3,4,6,7]. Our patient did not show any neurologic or psychiatric abnormality to date. Even though some children with type C NPD may present with visceral abnormalities only, the absences of neuropsychiatric symptoms make type C NPD less likely.

As with the case presented, painless abdominal distention due to hepatosplenomegaly is the most common presenting symptom in type B NPD. It is a presenting sign in about 90% of cases. This is a very important abnormality when seen in childhood because it predisposes patients to liver failure which is the first cause of death from this disease [3].

Our case had a history of cough, night sweats, and weight loss. These symptoms in combination with hepatosplenomegaly and the high burden of tuberculosis in our county, tuberculosis come at the top of the differential diagnosis. And most patients receive treatment for tuberculosis based on presentation alone even when laboratory tests are negative for tuberculosis, which is what happened with our case at the referring institution. The lungs are one of the commonly involved organs in all types of NPD, but presentation with pulmonary findings in childhood is highly suggestive of Type B. In a study of 53 patients with type B NPD, CT showed ILD in 98% percent of the patients. Ground-glass opacities, interlobular septal thickening, and intralobular lines were commonly seen findings, consistent with chest CT findings seen in our case. Pulmonary function tests are useful to evaluate the degree and assess the progression of the disease since imaging findings have a poor correlation with disease progression [9,10].

Bone marrow is another important target organ for deposition diseases. Hematologic abnormalities are one of the commonly encountered clinical and lab findings in patients with ASMD. Our patient presented with leukopenia, anemia, and thrombocytopenia. Studies have revealed that about 50% of patients with NPD have thrombocytopenia and about 20%-30% of patients have anemia and leukopenia [3].

Even though reduced activity ASM and genetic sequencing are confirmatory studies for ASMD, they are not available in our setup. Measurement of ASM activity from a dried blood spot [DBS] sample using recently developed commercial kits is enhancing first-line diagnosis. This kit measures ASM activity in DBS using tandem mass spectrometry [11].

Diagnosis of ASMD requires history, clinical examination, blood tests, sphingomyelinase enzyme activity in peripheral leukocytes, and genetic analysis. Histologic features seen on bone marrow, liver, or spleen also aid the diagnosis. Although sphingomyelinase enzyme activity in peripheral leukocytes and genetic analysis are diagnostic of ASMD, most developing countries, such as ours, still struggle to provide the facilities needed for such tests. Even though we were unable to do those diagnostic tests, clinical history, imaging finding of hepatosplenomegaly and ILD, pancytopenia in the lab and abundant foam cells on the liver and bone marrow are highly suggestive of type B chronic visceral form of ASMD.

So far, supportive care is the mainstay of treatment as there is no cure for NPD. Recent progress with enzyme replacement therapy with olipudase alfa, enzyme is promising for the treatment of non-neurologic manifestation of ASMD [11]. These treatments are currently unavailable in our country and are unaffordable to buy from abroad for most patients in our setting. Patient is currently lost from follow.

## Conclusion

Niemann-Pick disease is a rare lysosomal storage disease that exhibits variable clinical presentations, making it even more difficult to diagnose. A definite diagnosis can only be made by gene sequencing and measuring ASM activity. Unfortunately, in most of the developing countries, these studies are inaccessible. Physicians practicing in such a setup need to have a high index of suspicion. When more modern definitive diagnostic tools are unavailable, use of older method such as biopsied can help reach at a diagnosis and more importantly help in ruling out common diseases such as tuberculosis. In our case, the bone marrow and liver biopsy findings of foam cells helped in the diagnosis of NPD, almost certainly ASMD type B.

## Authors' contributions

All authors made a significant contribution to the work reported, whether that is in the conception, execution, acquisition of data, and interpretation, or in all these areas; took part in drafting, revising or critically reviewing the article; gave final approval of the version to be published; have agreed on the journal to which the article has been submitted; and agree to be accountable for all aspects of the work.

## Patient consent

A written informed consent was obtained from the patient's parents for publication of this case report and accompanying images. A copy of the written consent is available for review by the Editor-in-Chief of this journal upon request.
